# A tool to measure the influence of social media on health behaviors: an exploratory study

**DOI:** 10.1038/s41405-026-00417-0

**Published:** 2026-03-27

**Authors:** Chloé Rethaber, Clément Mathieu, Gabriel Fernandez de Grado, Damien Offner

**Affiliations:** 1 Faculté de Chirurgie Dentaire, Université de Strasbourg, Strasbourg, France; 2 Pôle de Médecine et Chirurgie Bucco-Dentaires, Hôpitaux Universitaires de Strasbourg (HUS), Strasbourg, France; 3 INSERM (French National Institute of Health and Medical Research), UMR 1260, Regenerative Nanomedicine (RNM)FMTS, Strasbourg, France

**Keywords:** Dental public health, Dental psychology

## Abstract

**Objectives:**

Social media is deeply embedded in our daily lives, particularly among younger generations. In the field of health, these platforms are both promising tools for prevention and channels for misinformation. This study aimed to develop and validate an exploratory measurement tool to quantify the influence of social media on health-related behaviors across three dimensions: social, economic, physical.

**Study design:**

Cross-sectional survey using a self-administered 15-item questionnaire.

**Methods:**

Items of the questionnaire were developed from literature and refined through expert consensus to cover social, economic, and physical dimensions of influence. Participants were voluntarily recruited in hospital waiting rooms in France; a subset completed the tool twice for reliability (test/retest). Analyses included Pearson correlations, Cronbach’s alpha, Intraclass Correlation Coefficients, and Multiple Correspondence Analysis to identify user profiles.

**Results:**

Data were collected from 110 participants aged 18 to 81. The instrument demonstrated excellent internal consistency (Cronbach’s alpha = 0.9), strong test-retest reliability (ICC = 0.93), and high score fidelity (Pearson *r* = 0.87). MCA revealed three user clusters: non-influenced older adults using Facebook, moderately influenced youth using Snapchat, and highly influenced young adults under 35 using Instagram and TikTok. Women and younger participants were more influenced economically and physically.

**Conclusions:**

This study highlights the growing influence of social media on health behaviors and introduces a reliable exploratory instrument to identify the most receptive populations. The tool can support targeted public health strategies and ethical engagement on digital platforms, especially for younger audiences frequently exposed to health-related content.

## Introduction

Nowadays, social media has become an essential part of our lifestyles. It is therefore important to explore the influence of social media on society, particularly in the field of health. Originally designed to connect individuals, social media has evolved into a powerful tool for communication and influence, giving rise to the profession of “influencers.”

In France, 92% of the population uses the Internet, and over 52 million people (i.e., 80%) are active on social media platforms [1]. In the United States, more than 97% of 13–17-year-olds use social media [2]. Platforms such as Facebook, Instagram, TikTok, and YouTube dominate, with preferences varying by generation [3, 4].

 Influencers play a key role in disseminating information and persuading internet users [5]. They are perceived as even more trustworthy when the information shared is simple and easy to understand [6], especially when they share content related to health topics [7]. Influencers are generally categorized into two main groups:

- Commercial influencers, who promote products and services, often through paid partnerships. Influencer marketing is booming globally, representing a multi-billion-dollar industry. More than 92% of influencers have collaborated with brands, whether paid or unpaid [8].
- Lifestyle influencers, who share advice and motivate their followers to adopt new habits [3].

Regarding health, although a growing number of professionals share reliable content on social media [9], most health content creators are not healthcare professionals, and health misinformation on these platforms is far from rare, posing significant risks [10–14]. Social media is a major source of health information, especially among adolescents [15, 16], who often highlight the feeling of anonymity and safety in accessing and sharing health-related content [11, 17]. This consumption of information can sometimes lead to an increased interest in cosmetic surgery [18] or the purchase of beauty products [14]. Therefore, it is essential for healthcare professionals to invest in social media to provide reliable prevention messages. When used wisely, social media can be a powerful vehicle for health promotion [11].

It should also be noted that while many young users seem increasingly aware of the presence of fake news on social media [19], verifying the truthfulness of information remains challenging due to the vast amount of content available [20]. This contributes to a disrupted perception of trust in health information shared online, as well as the degree to which users feel influenced by it.

Three main dimensions of influence can be highlighted:

- Social: Young people often prefer social media over traditional sources of information [15, 21].
- Economic: Digital marketing is transforming consumption patterns, using targeted strategies with measurable impacts on purchasing behaviors [14, 22].
- Physical: Social pressure related to appearance is leading more individuals to undergo cosmetic procedures and use beauty products influenced by social media [18, 21].

By exploring these social, economic, and physical dynamics, we aim to answer the question of how significantly social media influences users’ health behaviors. This research also addresses the lack of quantitative studies in the field of influence and trust in social media [11]. The exploratory measurement tool we have developed and validated to assess social media influence on health behaviors is aimed to be practically used at this stage and to help identify the most affected populations and guide strategies to maximize the positive impact of social media on public health. In doing so, social media could become an effective—and above all, targeted—lever for prevention.

## Methods

### Study design and participant recruitment

This study was conducted using an online questionnaire, made accessible through a dedicated platform. Participants were recruited on a voluntary basis. Posters containing a QR code linking directly to the questionnaire were displayed in the waiting rooms of a healthcare facility, specifically a hospital department of oral medicine and surgery in France, in order to reach a various panel of the population. A healthcare setting was chosen because it provided access to a heterogeneous population already familiar with health contexts, making it particularly relevant for investigating the influence of social media on health behaviors.

To ensure the reliability of the collected data and to assess the stability of responses over time, each participant was invited to complete the same questionnaire twice, with a two-week interval. To facilitate this follow-up, a personalized reminder email was sent to each participant two weeks after their initial participation.

To preserve anonymity, email addresses required for sending the second questionnaire were not collected through the main survey. Instead, a second QR code led to a separate page where participants could voluntarily submit their email address. This procedure ensured that no direct link could be made between participants’ responses and their identity.

### Ethics and consent

The study was approved by the Ethical Committee of the University Hospitals of Strasbourg, France, and received the authorization n°CE-2023-27. It was stated that implied consent was given by taking part in the study, as the link to the questionnaire

was freely available. Participants were informed at the beginning of the questionnaire of the purpose of the study, and that their participation would be voluntary and anonymous.

### Questionnaire design

The aim of the questionnaire was to measure the influence of social media on users in the field of health. It was designed to be completed twice, allowing for reliability assessment through a test/re-test procedure. An initial set of items was developed following ideas found in the literature and then discussed during a meeting that included a multidisciplinary panel: one general practitioner, two dentists, two public health specialists, and one practitioner specialized in pediatrics. This process led to consensus on the face and content validity of the items.

The questionnaire was then structured around three dimensions: social, economic, and physical. Each dimension included 5 questions, making a total of 15 items. Participants responded using a 4-point Likert scale with frequency options: never, less than once a month, one to two times a month, every week. The questions address various aspects of social media influence on health, including the use of health advice shared by influencers, the impact of this advice on personal and professional relationships, and tendencies to purchase health-related products or consult health professionals based on social media content.

To evaluate the influence across different population groups and based on the literature and conceptual framework of influence, we included demographic and behavioral determinants at the beginning of the questionnaire: age, gender, socio-professional category, and types of social media used. Participants who reported never using social media were excluded from the study.

Respondents were grouped into age categories reflecting their relationship to the internet and social media: under 25 years, 25–34 years, 35–54 years, and over 55 years.

The questionnaire items were, by dimension:

Social Dimension:

- I have clicked on a health-related post shared by a friend.
- I have subscribed to a health influencer following a recommendation or a friend’s post.
- I have used social media as a source of health information without verifying its accuracy.
- Health advice from an influencer has influenced my relationships with family and/or friends (either positively or negatively).
- Health advice from an influencer has influenced my professional relationships (either positively or negatively).

Economic Dimension:

- I have felt the urge to buy a health-related product after seeing a post from an influencer.
- I have been encouraged to purchase a health-related product after seeing a promotional code.
- The number of followers an influencer has, motivated me to purchase a health-related product (e.g., cream, pills, toothpaste, etc.).
- The connection between an influencer’s claimed profession and what they promote has influenced me to buy a health-related product.
- I have purchased a health-related product promoted or recommended by an influencer on social media.

Physical Dimension:

- I have consulted a healthcare professional after seeing a post on social media.
- I have followed/applied health advice shared by influencers.
- I use health or skincare products promoted or recommended by influencers.
- I have been influenced in my health-related decisions (e.g., vaccination, surgery, consultation with specialists) after reading a post or opinion on social media.
- I have considered undergoing esthetic medicine/surgery/dentistry to improve my appearance after seeing a post on social media.

### Influence measurement tool

The original response scale included four frequency options: never, less than once a month, one to two times a month, every week. A score from 0 to 3 was initially considered to reflect the proportional frequency of responses. However, for the sake of clarity and to enhance behavioral discrimination, results were ultimately analyzed using a dichotomous approach: responses of “never” and “less than once a month” were scored 0, while the other responses were scored 1. The maximum total score was therefore 15.

### Pre-test

A pre-test was conducted with 35 individuals, including healthcare professionals and laypeople, to ensure the clarity and relevance of the questionnaire. Participants provided feedback on their understanding and the appropriateness of the questions. One item related to anxiety levels was removed, as it was deemed off-topic or too intrusive.

### Public distribution

The questionnaire was offered to a voluntary sample of patients from the selected healthcare facility. It was distributed via posters placed in waiting rooms and flyers handed out to patients. These materials included two QR codes: the first directed users to the questionnaire, while the second allowed them to provide an email address to receive the follow-up link two weeks later—without compromising anonymity.

### Statistical analyses

Data collected through the online questionnaire were organized using Excel software. All statistical analyses were conducted using R software (version 4.3.2). Descriptive analyses were performed to characterize the studied variables. Results are presented as means and standard deviations for quantitative variables, and as frequencies and percentages for qualitative variables.

To identify social media user profiles and assess the impact of digital influence, a Multiple Correspondence Analysis (MCA) was conducted. MCA is a factorial analysis method used to explore associations between categorical variables, visualized through two- or three-dimensional maps. This approach helps reduce the variability in response profiles into synthetic quantitative dimensions, allowing easier graphical interpretation. Following the MCA, a hierarchical clustering analysis was performed to group individuals based on similarities and form distinct user clusters.

### Validation of the measurement tool

The tool’s validity was assessed through a test/re-test procedure [23] and the calculation of an intraclass correlation coefficient (ICC) to evaluate the reliability and stability of responses [24].

Reliability was further assessed using Pearson’s correlation coefficient. Lastly, internal consistency of the scale—confirming that the questions were useful and relevant—was evaluated using Cronbach’s alpha coefficient [25].

## Results

At the end of the study, data were collected from 110 participants. Among them, 28 completed the questionnaire twice, enabling test–retest reliability analysis. The sample displayed a wide age range (18–81 years) and included various socio-professional categories (Table 1).

**Table 1:** Sample description.

	**General population** *N* = 110
**Sex**^a^	
Male	40 (36,4)
Female	69 (62,7)
Not specified	1 (0,9)
**Age,** *in years*^a^	
Under 25 years	48 (43,6)
25 to 35 years	24 (21,8)
35 to 55 years	18 (16,4)
55 years and over	20 (18,2)
**Socio-professional category**^a^	
Artisan/Business owner	8 (7,3)
Executive and higher intellectual profession	15 (13,6)
Employee	25 (22,7)
Student	38 (34,5)
Manual worker	6 (5,5)
Intermediate profession	2 (1,8)
Retired	10 (9,1)
Unemployed	6 (5,5)
**Healthcare professional**^a^ - *Yes*	29 (26,4)
**Social network used**^a^	
Instagram	72 (65,5)
Snapchat	46 (41,8)
TikTok	40 (36,4)
Facebook	37 (33,6)
X	5 (4,5)
Linkedin	3 (2,7)
Other	4 (3,6)
*None*	3 (2,7)

^a^*n* (%).

### Validation of the measurement tool

The intraclass correlation coefficient (ICC) yielded values of 0.87 for individual reliability and 0.93 for the average ICC, indicating a high level of response reliability. An ICC close to 1 reflects strong similarity between values, thus confirming the stability of the results.

Pearson’s correlation coefficient was also 0.87, demonstrating strong test–retest reliability with a high positive correlation.

Finally, Cronbach’s alpha was 0.9, indicating excellent internal consistency and confirming that the questionnaire items were relevant and useful.

These results validated the measurement tool.

### Sample characteristics

The majority of participants were women (62.7%). The sample was relatively young, with more than 40% of participants under the age of 25. Approximately 18.2% reported low engagement with social media. One-third of respondents were students, and 26.4% worked in healthcare-related professions.

Regarding social media usage, over 65% used Instagram, more than 40% used Snapchat, and about one-third used either TikTok or Facebook (Table 1).

The three participants who reported not using any social media were excluded from the final analysis.

### Social dimension

In terms of social media participation, 29% of participants clicked on a health-related post shared by a friend at least once a month. Regarding subscriptions to influencers, 15.9% reported following a health influencer after a recommendation at least monthly. When it came to using social media as a source of health information, 20.6% did so at least once a month.

As for the influence of social media on personal relationships, 7.5% reported that influencer content had an impact on their family or friends at least once a month, while only 4 participants noted any influence in the professional sphere (Table 2).

**Table 2:** Answers to the questionnaire based on the 3 dimensions.

		Age				Sex	
	Total *N* = 107	Under 25 years *N* = 48	25 to 35 years *N* = 23	35 to 55 years *N* = 18	55 years and over *N* = 18	Men *N* = 40	Women *N *= 66
**Click on a post**							
Less than once a month	76 (71,0)	31 (64,6)	17 (73,9)	14 (77,8)	14 (77,8)	31 (77,5)	45 (68,2)
At least once a month	31 (29,0)	17 (35,4)	6 (26,1)	4 (22,2)	4 (22,2)	9 (22,5)	21 (31,8)
**Following an influencer**							
Less than once a month	90 (84,1)	38 (79,2)	20 (87,0)	14 (77,8)	18 (100,0)	36 (90,0)	54 (81,8)
At least once a month	17 (15,9)	10 (20,8)	3 (13,0)	4 (22,2)	0 (0,0)	4 (10,0)	12 (18,2)
**Use as a source of information**							
Less than once a month	85 (79,4)	36 (75,0)	16 (69,6)	17 (94,4)	16 (88,9)	35 (87,5)	50 (75,8)
At least once a month	22 (20,6)	12 (25,0)	7 (30,4)	1 (5,6)	2 (11,1)	5 (12,5)	16 (24,2)
**Influence on the family sphere**							
Less than once a month	99 (92,5)	43 (89,6)	21 (91,3)	17 (94,4)	18 (100,0)	38 (95,0)	61 (92,4)
At least once a month	8 (7,5)	5 (10,4)	2 (8,7)	1 (5,6)	0 (0,0)	2 (5,0)	5 (7,6)
**Influence on the professional sphere**							
Less than once a month	103 (96,3)	45 (93,8)	22 (95,7)	18 (100,0)	18 (100,0)	38 (95,0)	64 (97,0)
At least once a month	4 (3,7)	3 (6,3)	1 (4,3)	0 (0,0)	0 (0,0)	2 (5,0)	2 (3,0)
**Desire to purchase a product**							
Less than once a month	90 (84,1)	39 (81,3)	16 (69,6)	17 (94,4)	18 (100,0)	38 (95,0)	52 (78,8)
At least once a month	17 (15,9)	9 (18,8)	7 (30,4)	1 (5,6)	0 (0,0)	2 (5,0)	14 (21,2)
**Encouraged by promotional code**							
Less than once a month	97 (90,7)	42 (87,5)	20 (87,0)	17 (94,4)	18 (100,0)	40 (100,0)	57 (86,4)
At least once a month	10 (9,3)	6 (12,5)	3 (13,0)	1 (5,6)	0 (0,0)	0 (0,0)	9 (13,6)
**Encouraged by the number of followers**							
Less than once a month	102 (95,3)	45 (93,8)	22 (95,7)	17 (94,4)	18 (100,0)	39 (97,5)	63 (95,5)
At least once a month	5 (4,7)	3 (6,3)	1 (4,3)	1 (5,6)	0 (0,0)	1 (2,5)	3 (4,5)
**Encouraged by the profession**							
Less than once a month	99 (92,5)	44 (91,7)	20 (87,0)	17 (94,4)	18 (100,0)	39 (97,5)	60 (90,9)
At least once a month	8 (7,5)	4 (8,3)	3 (13,0)	1 (5,6)	0 (0,0)	1 (2,5)	6 (9,1)
**Purchase of care products**							
Less than once a month	104 (97,2)	47 (97,9)	22 (95,7)	17 (94,4)	18 (100,0)	40 (100,0)	64 (97,0)
At least once a month	3 (2,8)	1 (2,1)	1 (4,3)	1 (5,6)	0 (0,0)	0 (0,0)	2 (3,0)
**Seeking advice from a healthcare professional**							
Less than once a month	101 (94,4)	43 (89,6)	23 (100,0)	17 (94,4)	18 (100,0)	40 (100,0)	61 (92,4)
At least once a month	6 (5,6)	5 (10,4)	0 (0,0)	1 (5,6)	0 (0,0)	0 (0,0)	5 (7,6)
**Following influencer advice**							
Less than once a month	95 (88,8)	42 (87,5)	18 (78,3)	17 (94,4)	18 (100,0)	39 (97,5)	56 (84,8)
At least once a month	12 (11,2)	6 (12,5)	5 (21,7)	1 (5,6)	0 (0,0)	1 (2,5)	10 (15,2)
**Use of care products**							
Less than once a month	100 (93,5)	44 (91,7)	21 (91,3)	17 (94,4)	18 (100,0)	39 (97,5)	61 (92,4)
At least once a month	7 (6,5)	4 (8,3)	2 (8,7)	1 (5,6)	0 (0,0)	1 (2,5)	5 (7,6)
**Influence of health-related decisions**							
Less than once a month	102 (95,3)	45 (93,8)	22 (95,7)	17 (94,4)	18 (100,0)	39 (97,5)	63 (95,5)
At least once a month	5 (4,7)	3 (6,3)	1 (4,3)	1 (5,6)	0 (0,0)	1 (2,5)	3 (4,5)
**Thinking about esthetic medicine**							
Less than once a month	94 (87,9)	39 (81,3)	19 (82,6)	18 (100,0)	18 (100,0)	35 (87,5)	58 (87,9)
At least once a month	13 (12,1)	9 (18,8)	4 (17,4)	0 (0,0)	0 (0,0)	5 (12,5)	8 (12,1)

### Economic dimension

Around 15% of participants were influenced by social media to purchase health-related products, with women and those under 35 being more susceptible. The use of promotional codes was more common among women.

Actual purchases of healthcare or wellness products via social media were rare, with only 3 participants reporting such purchases at least once a month (Table 2).

### Physical dimension

Regarding consultation with healthcare professionals, 5.6% of respondents reported doing so at least once a month following a social media post. Approximately 11.2% reported following influencer advice on health at least once a month, with women more likely to do so.

Use of health or wellness products promoted on social media remained low, with only five participants influenced to use such products monthly or more.

About 10% of participants, especially those under 35, reported thinking about undergoing esthetic medicine or surgery after seeing related content on social media (Table 2).

### Healthcare professionals

More than half of the healthcare professionals reported clicking on health-related posts at least once a month.

Around 13.8% acknowledged being professionally influenced by social media content. These professionals were more likely to pay attention to the declared profession of the influencers.

In addition, 13.8% reported being personally influenced regarding their own health by social media— more than non-healthcare professionals (see supplementary material 1).

### Multiple Correspondence Analysis (MCA)

Multiple Correspondence Analysis is a statistical technique used to identify links between different variables in a dataset [26].

This method represents participants in a multidimensional space, with each axis corresponding to a variable.

Our study focused on the first two axes to model participants in 2D and identify discriminant patterns.

1. Variable Contributions:
  Economic variables and influencer health advice contributed heavily to the first axis.
  Age and type of social media used defined the second axis.
  Social media platform and gender were not discriminant on the first axis, but certain social and physical variables were on the second axis.
2. Graphical Interpretation:
  The graphical representation (Fig. 1) shows that participants influenced by social media in the physical or economic dimension cluster on the far right of the factorial map.

**Fig. 1:**
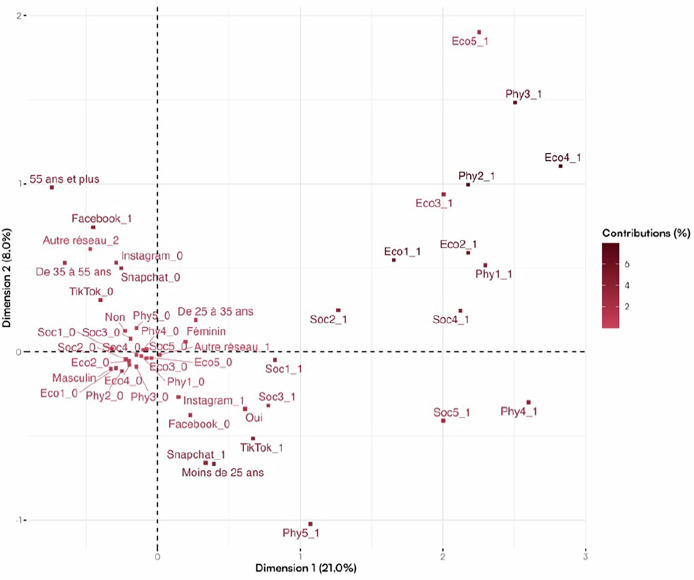
Representation of modalities on the first factorial plane. **Social dimension:** Soc1 click on a post; Soc2 following an influencer; Soc3 use as a source of information; Soc4 Influence on the family sphere; Soc5 Influence on the professional sphere. **Economic dimension:** Eco1 Desire to purchase a product; Eco2 Encouraged by a promotional code; Eco3 Encouraged by the number of followers; Eco4 Encouraged by the influencer’s profession; Eco5 Purchase of care products. **Physical dimension:** Phy1 Seeking advice from a health professional; Phy2 Following influencer’s advice; Phy3 Use of care products; Phy4 Influence on health-related choices; Phy5 Considering esthetic medicine.
  Participants under 25, using Snapchat and TikTok, considered social media a valid source of health information.
  Participants aged 35 and older, mainly Facebook users, were generally not influenced by social media.
3. Clustering:
  Three main clusters were identified (Fig. 2):
  Cluster 1: Uninfluenced social media users, mainly men aged 35+, Facebook users.

**Fig. 2:**
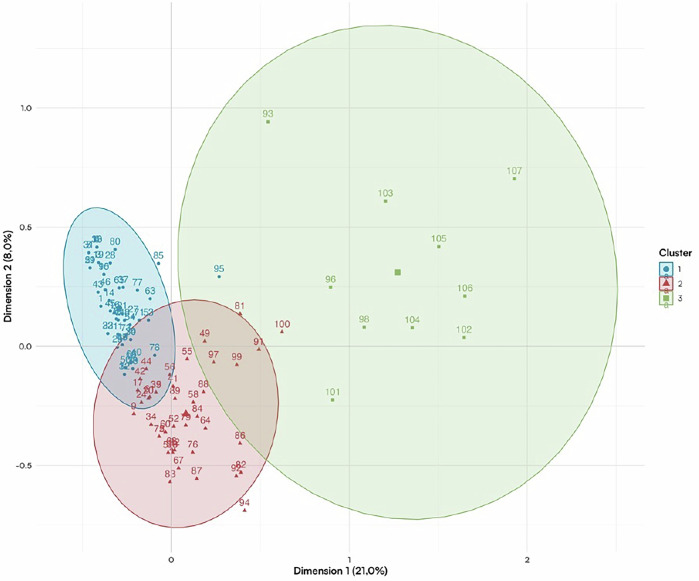
Clusters representation.
  Cluster 2: Snapchat users, mostly under 25, moderately influenced.
  Cluster 3: Heavily influenced users, mainly under 35, using Instagram and TikTok, influenced by promotional content and influencer advice.

The MCA revealed distinct profiles of social media users with varying levels of influence. Economic variables and influencer recommendations played a crucial role in shaping these profiles. Younger users were more likely to be influenced by social media, while older users were less affected. Details of these clusters are presented in Table 3.

**Table 3:** Results according to the three identified clusters.

	**Cluster**		
	**1** *N* = 55	**2** *N* = 42	**3** *N* = 10
**Score**^a^	0,4 (0,8)	1,2 (1,3)	6,2 (2,3)
**Sex**^b^ **-** *Female*	29 (52,7)	29 (69,0)	9 (90,0)
**Age**^b^, *in years*			
Under 25 years	8 (14,5)	34 (81,0)	6 (60,0)
25 to 35 years	11 (20,0)	8 (19,0)	4 (40,0)
35 to 55 years	17 (30,9)	0 (0,0)	0 (0,0)
55 years and over	19 (34,5)	0 (0,0)	0 (0,0)
**Halthcare professional**^b^ - *Yes*	6 (10,9)	17 (40,5)	6 (60,0)
**Social network used**^b^			
Facebook	30 (54,5)	5 (11,9)	1 (10,0)
TikTok	6 (10,9)	26 (61,9)	8 (80,0)
Snapchat	9 (16,4)	32 (76,2)	5 (50,0)
Instagram	27 (49,1)	36 (85,7)	8 (80,0)
Other	3 (5,5)	0 (0,0)	0 (0,0)
**Social dimension**^b^ - *Yes*			
Click on a post	8 (14,5)	14 (33,3)	8 (80,0)
Following an influencer	3 (5,5)	7 (16,7)	6 (60,0)
Use as a source of information	5 (9,1)	12 (28,6)	4 (40,0)
Influence on the family sphere	0 (0,0)	3 (7,1)	4 (40,0)
Influence on the professional sphere	0 (0,0)	2 (4,8)	2 (20,0)
**Economic dimension**^b^ - Yes			
Desire to purchase a product	1 (1,8)	7 (16,7)	8 (80,0)
Encouraged by promotional code	0 (0,0)	2 (4,8)	7 (70,0)
Encouraged by the number of followers	1 (1,8)	1 (2,4)	2 (20,0)
Encouraged by the profession	0 (0,0)	0 (0,0)	7 (70,0)
Purchase of care products	0 (0,0)	0 (0,0)	2 (20,0)
**Physical dimension**^b^ - Yes			
Seeking advice from a healthcare professional	0 (0,0)	2 (4,8)	3 (30,0)
Following influencer advice	1 (1.8)	1 (2.4)	9 (90.0)
Use of care products	0 (0,0)	1 (2,4)	5 (50,0)
Influence of health-related decisions	0 (0,0)	1 (2,4)	3 (30,0)
Thinking about esthetic medicine	0 (0,0)	9 (21,4)	4 (40,0)

^a^Mean (standard deviation).

^b^*n* (%).

## Discussion

### Biases

This study presents several potential biases. A selection bias is likely, as participants responded to an online questionnaire, which may exclude those less comfortable with digital tools. A self-selection bias is also present, as participants were recruited through posters in waiting rooms. Moreover, this was a single-center study, introducing a center effect bias. We also acknowledge that recruiting participants in a healthcare setting may have introduced a selection bias, as this population is already engaged with health services. Nevertheless, the sample remained heterogeneous and allowed us to validate the instrument in a relevant context. Although healthcare professionals and students were somewhat overrepresented, they represent a substantial share of the active population (e.g., approximately 7% in France [27] and 14% in the United States [28]) and their inclusion provides meaningful insights into the influence of social media on health behaviors. Indeed, they are themselves frequent consumers of health-related content on social media, making their perspectives relevant to the analysis. Future studies should extend recruitment to community, educational, or online settings to broaden representativeness and confirm the tool’s applicability across wider populations.

Another limitation of this study is that it did not follow all the conventional steps of scale development, such as exploratory and confirmatory factor analyses on independent samples. However, the items were generated from a literature review and refined through discussion and consensus with a multidisciplinary expert panel, which provided face and content validity. In addition, the tool demonstrated excellent internal consistency and strong test–retest reliability, offering preliminary evidence of its robustness. Importantly, by making this exploratory instrument available at this stage, we aim to provide a resource with practical usability for researchers and practitioners interested in measuring the influence of social media on health behaviors. This first version may serve as a foundation for future refinements, larger-scale validations, and international comparisons. It is also to note that our sample only contained 28 double-respondents for the test–retest and this should lead to the results being considered with caution.

Future research should aim to replicate these findings in larger and more diverse samples, including multicenter studies, in order to conduct exploratory and confirmatory factor analyses on independent populations and to further examine construct validity. Longitudinal and interventional studies would also be valuable to assess the tool’s responsiveness and practical relevance in evaluating public health actions conducted through social media. Through these successive steps, the instrument could progressively move toward full validation and broader applicability in research and public health contexts.

### Strengths

This study addresses an innovative topic with limited existing literature. To our knowledge, no prior tool has been developed to measure the perceived influence of social media on health behaviors, which led us to design a specific instrument for this study. Furthermore, this work responds to the lack of quantitative studies in the field [11], and the measurement tool we developed may enable comparisons not only across different user profiles within the same country but also internationally.

The sample is diverse, including participants aged 18–81, with a balanced distribution of gender and socio-professional categories. The validation of the questionnaire, at this exploratory stage, was ensured through repeated responses and systematic follow-up. Standardization of procedures by a single investigator reinforced the consistency and reliability of the results.

### Interpretation of results

Statistical tests confirmed the reliability of the questionnaire. The use of dichotomous scoring allowed for clear distinctions between participants who were influenced or not by social media. No significant differences were found by sex or age within the social dimension (data not displayed).

However, women and young participants (18–25 years old) were more economically influenced, especially regarding the purchase of health-related products. These findings are in line with a 2020 study on typical online shopper profiles [29], which showed that individuals classified as “shopping addicts”—averaging 19 online purchases per year—are predominantly women (61%).

In the physical dimension, women were more likely to follow influencer advice, particularly concerning esthetic medicine. Interestingly, healthcare professionals appeared more influenced by social media content related to their profession or personal health. One possible explanation is their professional environment, which may lead them to consume more health-related content, often shared by peers rather than by non-professionals. However, this hypothesis could not be verified within our study.

The multiple correspondence analysis identified three distinct groups:

First, the least influenced individuals—those over 35, mainly using Facebook. This result aligns with current trends, as Facebook is now rarely used by content creators [8]. Second, moderately influenced individuals—mostly under 25 and using Snapchat. Finally, the most influenced group—people under 35 using Instagram and TikTok. These platforms are highly conducive to influencer marketing [8], offering short-form video content that appeals to younger audiences [6] and utilizes algorithms to amplify popular content, thereby increasing consumer desire [30].

The use of MCA helped reveal how social media influence varies across demographic groups. This approach distinguished user behaviors and susceptibility based on age, gender, and platform usage. These insights may inform not only targeted marketing strategies but also public health policies.

### Comparison with the literature and future perspectives

Our study found that the most used platforms among participants were Instagram, Snapchat, and TikTok, followed by Facebook. A 2023 study by Reech [3] reported the same mostly used social media, although Facebook ranked first in that survey. This discrepancy is likely due to our younger sample, as Facebook is more commonly used by older demographics, whereas Instagram dominates among those under 35—a pattern also observed among young users in the United States [2].

Social media influences users differently depending on the population group, with a greater impact observed in specific subgroups. While health-related influence may appear limited compared to other areas like fashion or beauty [18, 21, 22], this is likely due to the relatively low number of health-focused content creators compared to the abundance of lifestyle influencers. Nonetheless, the risks of misinformation are significant. In particular, individuals who are highly influenced by poor-quality information may face serious consequences—for example, in the context of dental tourism.

Cost savings, promises of rapid esthetic transformation, and the influence of celebrities and social media platforms—especially TikTok and Instagram—play a central role in promoting dental tourism, particularly among younger audiences. Despite the glamorous portrayal of results, this trend raises concerns among healthcare professionals [31]. Many practitioners criticize the sometimes insufficient quality of care abroad, the lack of postoperative follow-up, and limited legal recourse in case of complications. Some dentists are even hesitant to treat returning patients due to medico-legal liability risks [10, 31].

Another important aspect to consider is digital health literacy, which has been shown to play a central role in shaping how children and young people engage with health information online [32]. It is defined as the ability to access, appraise, understand and apply health information to address a health problem [33]. Social media platforms are now a major source of health-related content, but the ability to critically appraise such information varies significantly across age, socioeconomic, and educational groups [34]. In our study, some groups were more frequently influenced by social media, particularly in the economic and physical dimensions. It is plausible that differences in digital health literacy partly explain this heightened vulnerability: individuals with lower skills in evaluating online information may be more exposed to persuasive marketing strategies and health-related misinformation.

Future research should therefore explore the intersection between digital health literacy and social media influence more systematically, may be by using the measurement tool we have developed in this study. Doing so could help identify protective factors that enable certain groups to resist misleading or harmful content, while highlighting at-risk populations who might benefit from tailored interventions. From a public health perspective, initiatives aiming to improve digital health literacy—such as school-based curricula, targeted campaigns for young adults co-designed with young people and health providers [32] —could strengthen individuals’ ability to navigate online health environments. By addressing this underlying determinant, it may be possible to reduce inequalities in access to reliable information and to foster healthier, more informed decision-making across diverse demographic groups.

Regulatory initiatives have been introduced to act as a form of oversight over social media platforms. In several countries, such as France and Australia, access to social media is prohibited below the age of 15 or 16; in France, commercial posts must clearly disclose their promotional nature, and retouched photographs used in advertising must indicate that they have been altered [35, 36]. However, these regulations remain general in scope and do not specifically target health-related content. It may be regrettable that, in doing so, they also limit younger users’ access to high-quality preventive content that could be shared by healthcare professionals. In addition, private initiatives have attempted to emerge, particularly those aimed at certifying health-related accounts [37]. Yet, lacking institutional and/or governmental support, they have not been able to establish themselves sustainably. It will be interesting to observe the future measures proposed to protect populations from harmful health-related messages on social media.

Considering these elements, and the biases previously outlined, our study suggests that social media platforms such as Instagram and TikTok could serve as powerful tools for health prevention targeting people under 35—especially women—due to their high engagement with social media and greater receptivity to online information. These platforms could be used to promote healthier habits and encourage regular medical consultations, provided that healthcare professionals adopt the same influential codes as successful content creators. These rely on several key psychological principles [38]: reciprocity, likability, social proof, authority, scarcity, commitment, and unity. These mechanisms help influencers capture attention and shape user behavior.

Healthcare professionals on social media can help improve the quality of health information and prevention, in contrast to the sometimes questionable or even dangerous content shared by certain influencers [10]. However, depending on national laws, their presence online can raise ethical and professional concerns—particularly regarding self-promotion and patient acquisition [39, 40].

Furthermore, the measurement tool developed in this study can be used over time to monitor whether populations continue to be influenced by social media. This could help identify target groups for health campaigns and tailor messages to improve the effectiveness of public health strategies deployed on social platforms.

## Conclusion

The results of this study highlight the potential of social media as a tool for health prevention, especially among younger generations. Platforms such as Instagram and TikTok, widely used by individuals under 35, offer unique opportunities to disseminate health messages and promote responsible behaviors, as this group appears particularly susceptible to digital influence. However, the influence of social media must be regulated to avoid the spread of misinformation. Healthcare professionals have a central role to play in ensuring the quality and reliability of the content shared online.

It is therefore crucial to rely on the receptiveness of different population segments to develop effective prevention strategies. The measurement tool created and validated through this study is a novel instrument that enables the identification of target audiences and the adaptation of messages accordingly—thereby maximizing the impact of public health initiatives on social media. This research underscores the importance of developing ethical and tailored approaches to fully leverage the potential of digital tools in healthcare.

## Supplementary information

Supplemental Material File #1

## Data Availability

The data that support the findings of this study are available upon reasonable request from the authors.
