# Supplementary material for: A tool to measure the influence of social media on health behaviors: an exploratory study
Source: BDJ Open. 2026 Mar 27;12:26. doi: 10.1038/s41405-026-00417-0 (PMC13031869; doi:10.1038/s41405-026-00417-0)

- Supplementary material 1: Statistical analysis by healthcare professional status

|  |  | **No**  N = 79 | **Yes**  N = 29 |
| --- | --- | --- | --- |
| **Social dimension** |  |  |  |
| **Click on a post** *1* |  |  |  |
| Less than once a month | 77 (71,3) | 63 (79,7) | 14 (48,3) |
| At least once a month | 31 (28,7) | 16 (20,3) | 15 (51,7) |
| **Following an influencer***1* |  |  |  |
| Less than once a month | 91 (84,3) | 69 (87,3) | 22 (75,9) |
| At least once a month | 17 (15,7) | 10 (12,7) | 7 (24,1) |
| **Use as a source of information***1* |  |  |  |
| Less than once a month | 86 (79,6) | 64 (81,0) | 22 (75,9) |
| At least once a month | 22 (20,4) | 15 (19,0) | 7 (24,1) |
| **Influence on the family sphere** *1* |  |  |  |
| Less than once a month | 100 (92,6) | 75 (94,9) | 25 (86,2) |
| At least once a month | 8 (7,4) | 4 (5,1) | 4 (13,8) |
| **Influence on the professional sphere** *1* |  |  |  |
| Less than once a month | 104 (96,3) | 79 (100,0) | 25 (86,2) |
| At least once a month | 4 (3,7) | 0 (0,0) | 4 (13,8) |
| **Economic dimension** |  |  |  |
| **Desire to purchase a product** *1* |  |  |  |
| Less than once a month | 91 (84,3) | 68 (86,1) | 23 (79,3) |
| At least once a month | 17 (15,7) | 11 (13,9) | 6 (20,7) |
| **Encouraged by promotional code** *1* |  |  |  |
| Less than once a month | 98 (90,7) | 74 (93,7) | 24 (82,8) |
| At least once a month | 10 (9,3) | 5 (6,3) | 5 (17,2) |
| **Encouraged by the number of followers** *1* | | | |
| Less than once a month | 103 (95,4) | 75 (94,9) | 28 (96,6) |
| At least once a month | 5 (4,6) | 4 (5,1) | 1 (3,4) |
| **Encouraged by the profession** *1* |  |  |  |
| Less than once a month | 100 (92,6) | 76 (96,2) | 24 (82,8) |
| At least once a month | 8 (7,4) | 3 (3,8) | 5 (17,2) |
| **Purchase of care products** *1* |  |  |  |
| Less than once a month | 105 (97,2) | 77 (97,5) | 28 (96,6) |
| At least once a month | 3 (2,8) | 2 (2,5) | 1 (3,4) |
| **Physical dimension** |  |  |  |
| **Seeking advice from a healthcare professional***1* | | | |
| Less than once a month | 102 (94,4) | 75 (94,9) | 27 (93,1) |
| At least once a month | 6 (5,6) | 4 (5,1) | 2 (6,9) |
| **Following influencer advice***1* |  |  |  |
| Less than once a month | 96 (88,9) | 73 (92,4) | 23 (79,3) |
| At least once a month | 12 (11,1) | 6 (7,6) | 6 (20,7) |
| **Use of care products***1* |  |  |  |
| Less than once a month | 101 (93,5) | 75 (94,9) | 26 (89,7) |
| At least once a month | 7 (6,5) | 4 (5,1) | 3 (10,3) |
| **Influence of health-related decisions***1* |  |  |  |
| Less than once a month | 103 (95,4) | 78 (98,7) | 25 (86,2) |
| At least once a month | 5 (4,6) | 1 (1,3) | 4 (13,8) |
| **Thinking about aesthetic medicine***1* |  |  |  |
| Less than once a month | 95 (88,0) | 71 (89,9) | 24 (82,8) |
| At least once a month | 13 (12,0) | 8 (10,1) | 5 (17,2) |
| *1* n (%) *; 2* Chi-2 test, exact Fisher test |  |  |  |

- Supplementary material 2: Contribution of variables by axis

***Social Dimension***

***Soc1*** *Click on a post* ***Soc2*** *Following an influencer*

***Soc3*** *Use as a source of information* ***Soc4*** *Influence on the family sphere*

***Soc5*** *Influence on the professional sphere*

***Economic Dimension***

***Eco1*** *Desire to purchase a product* ***Eco2*** *Encouraged by a promotional code*

***Eco3*** *Encouraged by the number of followers* ***Eco4*** *Encouraged by the influencer's profession* ***Eco5*** *Purchase of care products*

***Physical Dimension***

***Phy1*** *Seeking advice from a health professional* ***Phy2*** *Following influencers' advice*

***Phy3*** *Use of care products* ***Phy4*** *Influence on health-related choices*

***Phy5*** *Considering aesthetic medicine*


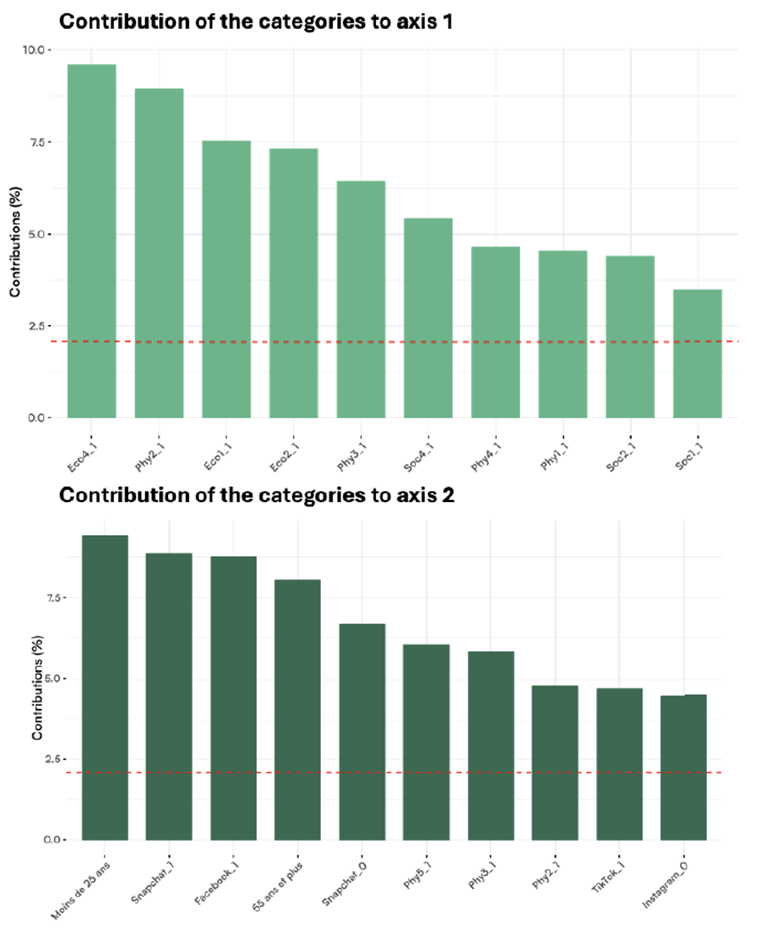

Supplement: Supplementary file 1 — Supplemental Material File #1 [file 41405_2026_417_MOESM1_ESM.docx]
